# Heart Rate and Heart Rate Variability of Rhesus Macaques (*Macaca mulatta*) Affected by Left Ventricular Hypertrophy

**DOI:** 10.3389/fvets.2019.00001

**Published:** 2019-01-22

**Authors:** Yu Ueda, Taylor L. Slabaugh, Ashley L. Walker, Eric S. Ontiveros, Paul-Michael Sosa, Rachel Reader, Jeffrey A. Roberts, Joshua A. Stern

**Affiliations:** ^1^Department of Medicine & Epidemiology, School of Veterinary Medicine, University of California, Davis, Davis, CA, United States; ^2^California National Primate Research Center, University of California, Davis, Davis, CA, United States

**Keywords:** hypertrophic cardiomyopathy, sudden death, arrhythmia, autonomic balance, electrocardiography, Holter analysis

## Abstract

Hypertrophic cardiomyopathy (HCM) is frequently associated with sudden cardiac death, presumably due to the development of malignant arrhythmias. The risk of sudden cardiac death due to HCM has been reported to be predicted by assessing electrocardiographic (ECG) changes including frequencies and complexities of arrhythmias as well as heart rate variability (HRV) as an assessment of autonomic balance. Sudden cardiac death in association with naturally-occurring left ventricular hypertrophy (LVH) has been reported in a colony of rhesus macaques and is under investigation as a potential non-human primate model of human HCM. In the present study, 10 rhesus macaques with LVH and 10 without the signs of LVH confirmed by an echocardiographic examination were recruited for assessing ECG and HRV parameters. ECG morphology on 10-s, 6-lead ECG analysis, and the frequency and complexity of arrhythmias as well as HRV on 20-h ambulatory ECG Holter analyses were assessed. On the standard 10-s 6-lead ECG analysis, P wave and QRS complex duration as well as the QRS complex amplitude were significantly increased in the LVH-affected rhesus macaques compared to control rhesus macaques. Analysis of 20-h Holter monitoring revealed no statistically significant differences in the frequency or the complexity of arrhythmias between the LVH and the control groups. Several HRV parameters were smaller in the LVH group than the control group throughout the majority of Holter recordings showing periods of reduced variability, however, no statistically significant differences were achieved across groups and/or time points. These findings indicate that ECG analysis and Holter monitoring of rhesus macaques are feasible and that ECG morphological changes in association with LVH could be used as a possible component of an antemortem screening tool. The rhesus macaques of this study did not reveal clear indications of risk for sudden cardiac death. Further studies are necessary to determine the etiology of sudden cardiac death due in LVH affected rhesus macaques and identify if any parameters of arrhythmia assessment or HRV can be used to predict the development of sudden cardiac death.

## Introduction

Hypertrophic cardiomyopathy (HCM) is associated with various clinical consequences ranging from no apparent symptoms to the development of congestive heart failure, thromboembolic disease, arrhythmias, as well as sudden cardiac death (SD) (1, 2). HCM in people has been known to be associated with developing sustained ventricular and supraventricular arrhythmias (3), and thus the mechanisms of SD in humans, especially children, and young adults with HCM, is primarily attributed to the presence of severe arrhythmias (1, 4, 5). In addition to arrhythmias, the majority of human patients with HCM have documented abnormal electrocardiographic (ECG) patterns and morphologies, even before developing detectable structural ventricular change (6, 7).

Naturally-occurring inherited left ventricular hypertrophy (LVH) has been identified in a colony of rhesus macaques housed at the California National Primate Research Center (CNPRC) over the last two decades (8). Among these cases, SD has been the first and only clinical manifestation identified. Preliminary pathological and pedigree investigations of these rhesus macaques highlighted similar disease features when compared to human familial HCM. These features include concentric hypertrophy of left ventricular walls with diminished left ventricular lumen size. Echocardiographic examination as a part of antemortem examination has also identified key features resembling human HCM with concentric hypertrophy of the left ventricle as well as the presence of diastolic dysfunction (9). Based on the similarities of these post-mortem and ante-mortem findings between HCM in humans and LVH in rhesus macaques, it is reasonable to predict the SD in the rhesus macaques with LVH are also associated with the development of malignant arrhythmic events. However, no previous study has been performed to identify the prevalence and severity of arrhythmias in rhesus macaques with LVH compared to healthy rhesus macaques.

Holter monitoring has been commonly used in both human and veterinary medicine to quantify and analyze the frequency and complexity of cardiac arrhythmias since it is expected to be more sensitive for detecting arrhythmias than short-term standard ECG analysis (10). Holter monitoring in human patients with HCM revealed atrial fibrillation and other supraventricular arrhythmias are the most common arrhythmia detected (3). Ventricular arrhythmias are less common than supraventricular arrhythmias in human HCM patients, but it has been identified as one of the major risk factors associated with SD (11). Holter monitoring has also been used to assess heart rate variability (HRV), which is altered based on physiologic conditions and disease states. Of these conditions, the autonomic nervous system has the largest influence on an alteration of HRV (12–14) and quantification of HRV has therefore been used to characterize the sympathetic and parasympathetic balance in patients with cardiac or systemic diseases. For example, reduced HRV was identified to be associated with higher mortality in people who have suffered a myocardial infarction and in those with heart failure (15–17). In people with HCM, several studies found conflicting results with both reduced and increased HRV previously reported in patients with HCM (18–21). In addition, some studies found different results between adults and children regarding HRV as a prognostic indicator; decreased HRV did not improve the risk stratification in adults with HCM, while reduced HRV could be predictive of a higher risk of SD in children with HCM (22, 23). HRV has never been assessed in rhesus macaques with cardiac disease, and might provide us another tool to assess their autonomic nervous system status and potentially assist in antemortem diagnosis and risk stratification for LVH and SD.

The objective of the present study is to compare the ECG patterns on standard 6-lead ECG, the frequency and complexity of ECG identified arrhythmias over 20 h, and HRV in rhesus macaques with and without LVH. We hypothesized that rhesus macaques with LVH would have ECG pattern abnormalities attributed to the hypertrophic ventricular walls, more frequent and complex cardiac arrhythmias as well as reduced HRV parameters when compared to control rhesus macaques.

## Materials and Methods

### Subjects and Housing

All study procedures were conducted at the CNPRC. This study was carried out in accordance with the recommendations of USDA Animal Welfare Act and the Guide for the Care and Use of Laboratory Animals (24). The protocol was approved by the Institutional Animal Care and Use Committee of the University of California-Davis. The animal care and use program at the CNPRC is USDA-registered and AAALAC-accredited. Animals enrolled in the present study were housed in individual indoor cages during the study period (24 h) until the electrocardiographic equipment was removed. Alternating 12-h periods of light and darkness, controlled temperature, humidity, and ventilation were provided during the study period. Commercial primate chow (LabDiet Monkey Diet 5047, Purina Mills International, St Louis, MO) twice daily supplemented with vegetables and fruits biweekly were provided. Water was provided *ad libitum* using automatic watering devices. Annual physical examination, weighing, tuberculosis testing, dental prophylaxis, and routine blood tests including serum biochemistry and complete blood count analyses were performed at least annually under sedation. All rhesus macaques were also monitored periodically for bacterial and viral infections (SIV, simian T-lymphotrophic virus, and simian type D retrovirus, and herpes B virus) (25).

### Sedation

Rhesus macaques were fasted overnight prior to sedation for the study protocols. Shortly prior to echocardiographic examination, each animal was sedated with ketamine hydrochloride (10 mg/kg IM; Ketaject, Phoenix Pharmaceutical, St Joseph, MO) for echocardiographic and electrocardiographic assessment as well as ambulatory ECG (Holter) device placement and jacketing. They were sedated again with the same dose of ketamine for Holter device removal 24–26 h after the initial placement of the Holter device.

### Echocardiographic Examination

The sedated rhesus macaques were positioned in right and left lateral recumbency consecutively for standard echocardiographic examination (Philips CX50 Ultrasound Machine, Philips U.S.A., Andover, Massachusetts). All echocardiographic screenings were performed by a board-certified veterinary cardiologist (JS) using a 4–12-mHz sector-array transducer (S12-4) with color, pulsed-wave and continuous-wave spectral Doppler capability. All measurements and phenotype classification (LVH positive vs. control) were completed using standard offline analysis software (Syngo Dynamics, Siemens, Erlangen, Germany). All measurements were conducted with the leading-edge to leading-edge method on at least three cardiac cycles (consecutive when possible) in accordance with the guidelines of the American Society of Echocardiography. Measurements were not performed on cycles following any observed cardiac arrhythmia.

**Figure 1:**
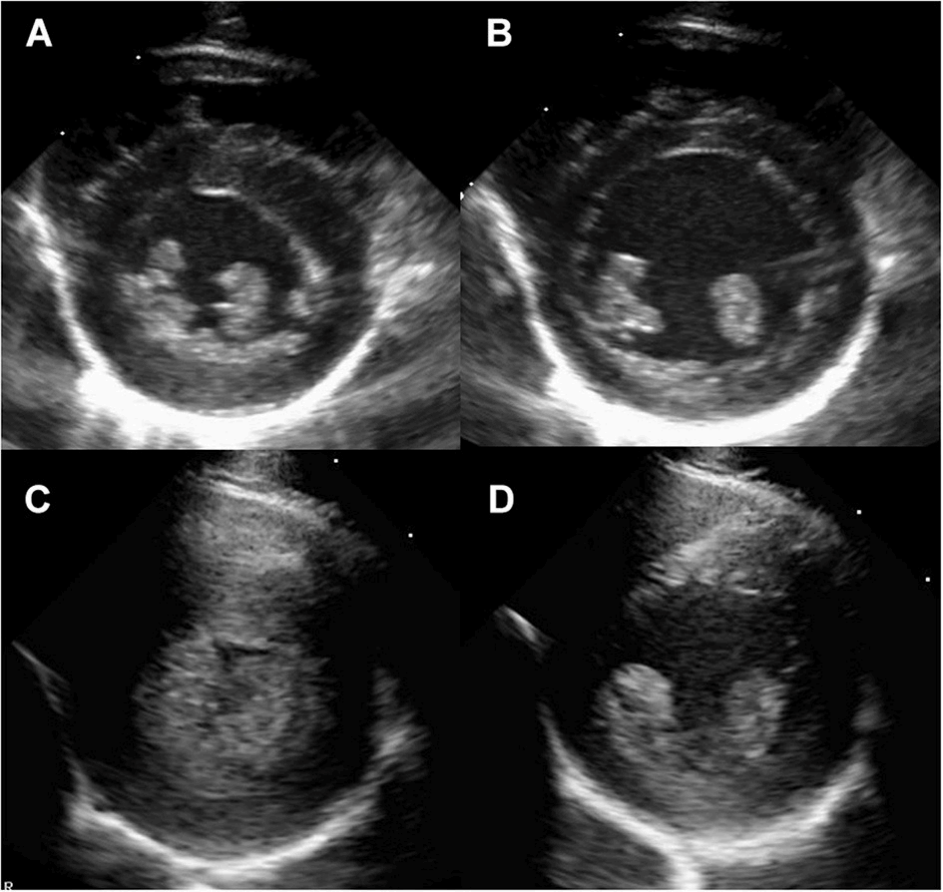
Right parasternal short axis imaging at the level of the papillary muscle in a normal rhesus macaque **(A,B)** and a rhesus macaque with severe left ventricular hypertrophy consistent with HCM **(C,D)**. The images are presented as systole **(A,C)** and diastole **(B,D)**.

The aortic root and left atrial diameters were obtained from the right parasternal short-axis and long-axis four-chamber two-dimensional views. From the right parasternal short-axis M-mode view, the interventricular septal wall thickness during diastole (IVSd) and left ventricular posterior wall thickness during diastole (LVPWd) were obtained at the chordae level (Figure 1). Transmitral peak flow velocities for passive filling and atrial contraction were measured from the left parasternal apical four-chamber view by using the pulsed-wave spectral Doppler image. The sample gate was placed at the tips of the mitral valve leaflets when they are wide open during diastole. Given that the parallel alignment of the sample gate was obtained in all rhesus macaques, no angle corrections were necessary. Color-tissue Doppler images were obtained at the septal and free-wall mitral annulus from the left apical four-chamber view, and peak velocities were measured in early and late diastole.

### Echocardiographic Diagnosis of LVH

Ten LVH-affected rhesus macaques were identified from the echocardiographic database developed by the author of the present study (JS). LVH in rhesus macaques was diagnosed by the combination of echocardiographic evidence of a thickened ventricular wall and the presence of diastolic dysfunction. Diagnosis of LVH was defined as a thickness of the LVPWd and/or IVSd exceeding 6.5 mm for rhesus macaques younger than 9 years old. Rhesus macaques older than or equal to nine years were diagnosed with LVH if IVSd was ≥8.8 mm and/or LVPWd was ≥7.4 mm (9). Diastolic dysfunction was diagnosed by pulsed-wave spectral Doppler trans-mitral filling patterns with a ratio of early to late filling velocities ≤ 0.9, or with the spectral tissue Doppler lateral or medial peak mitral annular velocity during early and late filling phase <0.9.

### Characteristics of Rhesus Macaques

Ten rhesus macaques that fulfilled the above criteria were enrolled in the present study as LVH-affected rhesus macaques. Ten clinically healthy rhesus macaques without LVH based on a normal echocardiographic examination were also enrolled in the study. These control rhesus macaques were matched to the LVH cases based on their age, sex, and body weight. Based upon pedigree assessment, the control rhesus macaques were not genetically related to the rhesus macaques with LVH within at least three generations.

### Standard 10 s 6-Lead Electrocardiographic Measurement

**Figure 2:**
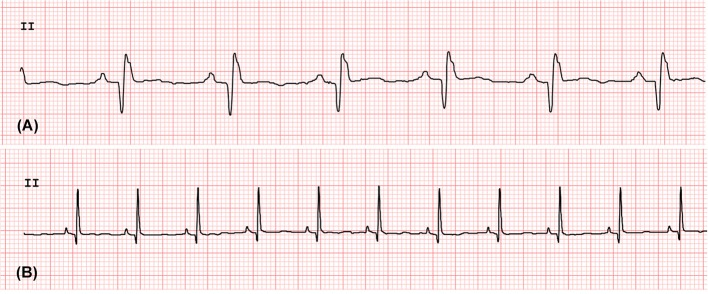
The standard 6-lead ECG waves obtained from the rhesus macaques with LVH **(A)** and without LVH **(B)**. The lead II strips were obtained at the paper speed of 50 mm/s and vertical ECG calibration of 20 mm/mV.

Following echocardiographic examination, rhesus macaques were placed in right lateral recumbency for ECG evaluation. Surface electrodes with flattened alligator clips were attached to the skin at the level of the elbows and stifles on the caudal aspect of the forelimbs and cranial aspect of the hindlimbs, respectively. Standard 6-lead ECGs (Philips PageWriter TC70 Cardiograph, Philips U.S.A., Andover, Massachusetts) were recorded for all rhesus macaques and a 10-s rhythm strip (leads I, II, III, aVF, aVL, aVR) was obtained at the paper speed of 50 mm/s and vertical ECG calibration of 20 mm/mV. Rhythm analysis was conducted by visual inspection. ECG measurement of the amplitude and duration of P waves, QRS complexes, as well as the duration of PQ and QT intervals were performed using standard, manual ECG calipers using lead II (Figure 2). Each measure was recorded for three consecutive complexes and averaged (avoiding any measures of complexes immediately preceding or following a noted cardiac arrhythmia). Heart rate was calculated based upon the average of all R to R interval measurements during the 10 s ECG rhythm strip. In addition, the mean electrical axis was calculated via standard methodology (26).

### Holter Device Placement and Recording

**Figure 3:**
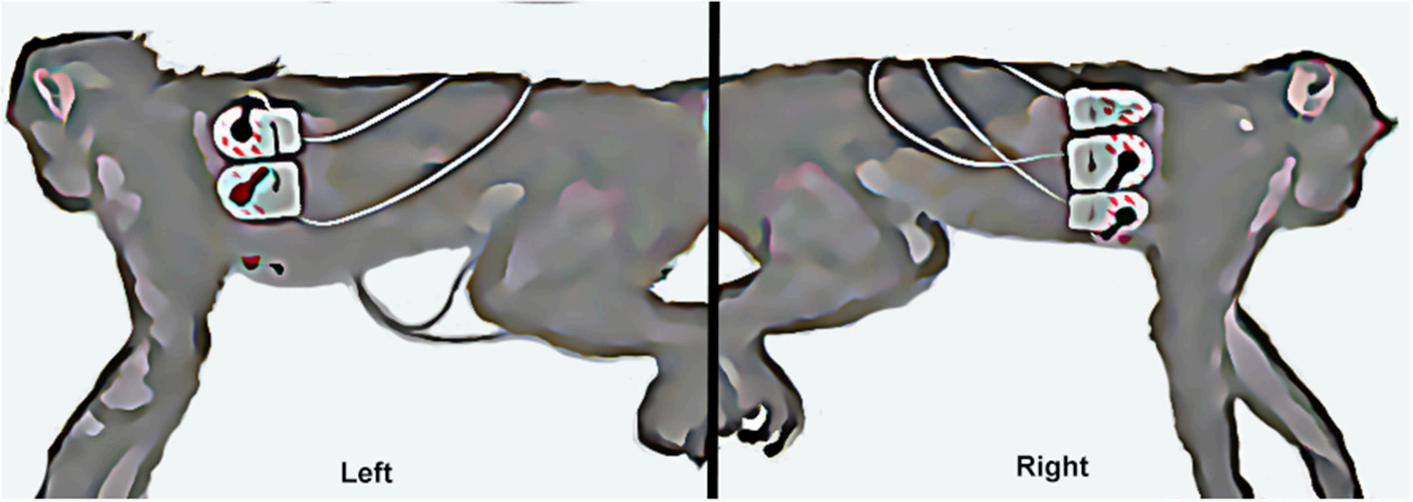
The **left** picture shows the left sided attachment of electrodes and ECG leads (left arm and left leg leads). The **right** picture shows the right sided attachment of electrodes and ECG leads (right arm, chest, and right leg leads).

After the short-term ECG examination, a 6 electrode 24-h ambulatory ECG device with three-channel recording and standard pacemaker spike detection (Burdick H3^+^ Holter Recorder, Mortara Instrument Inc. Milwaukee, WI) was attached in a standard precordial configuration to areas of the shaved chest that optimized the electrocardiographic signal (Figure 3). The device and bandage were also covered by a standard mesh primate jacket (Rhesus Primate Jackets, Harvard Apparatus U.K. Harvard Bioscience, Inc., Cambridge, U.K.) fitted over the device to prevent the rhesus macaques from accessing the wires and monitor. The rhesus macaques were individually confined within a cage located in a quiet indoor ward after recovering from sedation. Between 24 and 26 h after initiating the Holter monitoring, the rhesus macaques were again sedated with ketamine hydrochloride (10 mg/kg IM) for the removal of the jacket, bandage, and Holter device. After device removal the animals were monitored for recovery over 2 h and then returned to their routine housing.

### Arrhythmia and HRV Data Acquisition and Processing

All data from the Holter monitors were downloaded from the data storage card to the software analysis system, Vision 5 software (Mortara Instrument Inc., Milwaukee, WI). The downloaded data was randomized by one of the authors (EO) in order to provide a blinded data analysis performed by the authors (TS, YU) who were blinded to the animal identification and disease status of the rhesus macaques. The software analysis system automatically annotated normal and abnormal complexes, but all the complexes were prospectively, manually reviewed, and relabeled as needed to determine the frequency and complexity of ventricular or supraventricular ectopic beats and facilitate accurate analysis of HRV. The data with motion-related artifact was marked as artifact, discarded and not quantified. Due to the potentially significant effects of sedatives and stress from the Holter device placement, the first 4 h of the ECG recording were discarded, and the following 20-h ECG recordings were utilized for further analysis.

**Figure 4:**
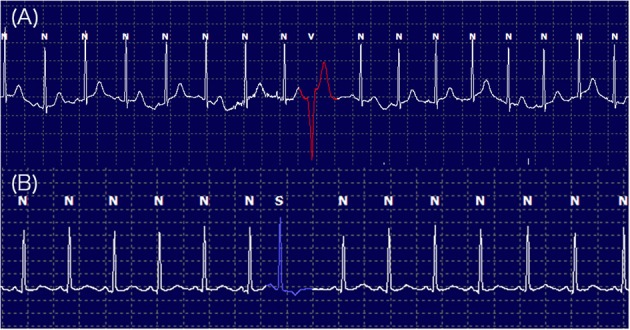
Twenty-hour Holter monitoring illustrating the frequencies and complexities of ventricular arrhythmia labeled as V **(A)** and supraventricular arrhythmia labeled as S **(B)** with normal QRS complexes labeled as N.

Supraventricular and ventricular arrhythmias were classified based on a complexity scale: 1 = single premature complexes, 2 = couplets, 3 = triplets, or 4 = consecutive (≥ 4 complexes) morphological abnormalities or ventricular tachycardia (Figure 4). Ventricular arrhythmias were classified based on the instantaneous heart rate (HR) as premature (HR ≥ 160), accelerated idioventricular (HR = 100–159), or escape (HR ≤ 99 bpm) complexes.

Heart rate variability was analyzed using standard time-domain techniques in accordance with published recommendations (27, 28). All of the time-domain measures of HRV over 20 h were calculated for each 1 h and averaged over the full 20 h using Vision 5 software. Each QRS complex was automatically detected and the normal-to-normal (NN) intervals were calculated from one R wave to the next R wave in the software. All QRS complexes were reviewed by the authors (YU, TS, EO) and the NN intervals were manually corrected as needed. Based on the NN intervals, the mean, minimal, and maximal HR were recorded. The standard deviation of all NN intervals (SDNN), the standard deviation of the average NN intervals over 5 min (SDANN), the square root of the mean squared differences of successive NN interval (RMSSD), and the number of interval differences of successive NN intervals >50 ms divided by the total number of NN intervals (pNN50) were obtained. In addition, the NN intervals were converted to the triangular index value that is the integral of the density distribution of the NN intervals divided by the maximal density distribution. All QT-intervals were also automatically measured, and the minimal, mean, and maximal QT-intervals were calculated and reported. To eliminate the effect of the HR on the QT-interval, the corrected QT-intervals (QTc) were calculated using the standard Bazett formula and used for the statistical analysis (29).

### Statistical Analysis

An a-priori sample size calculation was conducted based on the information from another HCM model species. Healthy humans are known to have a mean of 98 ventricular ectopic beats over 24 h and a standard deviation of 60 ventricular premature contractions (30). Humans with HCM are expected to have twice as many ectopic events compared to unaffected humans (31, 32). Thus, in our calculations we considered that rhesus macaques with LVH may also be expected to have at least twice as many ventricular ectopic beats when compared to control animals without LVH. A minimum of eight rhesus macaques in each group are needed to achieve 90% power with alpha of 0.05 and two-sided test. Given the possibility for intolerance to monitoring equipment, data loss due to electrical interference and other potential pitfalls, the sample size was increased to 10 rhesus macaques in each group.

Signalment (age, weight, gender), ECG amplitudes and intervals, frequencies of supraventricular and ventricular ectopic complexes, and the mean values of HR and HRV parameters in LVH and control groups were compared using either a non-paired Student's *t*-test (parametric) or Mann-Whitney test (non-parametric) after visual inspection and D-Agostino and Pearson Omnibus normality testing. The trend of the HRV parameters every 1-h over the course of 20 h was analyzed by performing repeated measures with a linear mixed model. For assessing correlations of HRV variables with selected demographic and echocardiographic parameters (IVSd, LVPWd, weight, and age), multivariate regression analysis was performed. A *P* < 0.05 was set as a threshold for the statistical significance. Statistical analysis was performed on commercially available GraphPad Prism software (GraphPad Prism v7.0c, La Jolla, CA) and STATA software (Stata Corporation v14.2, College Station, TX).

## Results

### Characteristics of Rhesus Macaques

The average weight was 11.52 kg (±4.07 kg) among all rhesus macaques, and 9.71 kg (±3.03 kg) and 13.34 kg (±4.30 kg) in the control and LVH-affected groups, respectively. The average age among all rhesus macaques was 10.4 (±3.71) years old, and 9.75 (±3.80) years old and 11.1 (±3.68) years old in control and LVH-affected groups, respectively. A total of ten males and ten female rhesus macaques were enrolled in the present study. Five females and five males were enrolled in the control and LVH groups. No statistically significant differences were identified in body weight, age, or sex between the control and LVH groups. Echocardiographic examination performed prior to ECG examination and placement of the Holter monitoring device confirmed the presence of LVH and absence of any other significant cardiac abnormalities. The average IVSd and LVPWd were 0.464 ± 0.084 cm and 0.492 ± 0.065 cm in the control group, while 0.696 ± 0.15 cm and 0.851 ± 0.16 cm in the LVH group. All rhesus macaques in the LVH group showed echocardiographic findings compatible with diastolic dysfunction, whereas no rhesus macaques in the control group had diastolic dysfunction.

### Analysis of 10 s 6-Lead ECG Measurements

Standard 6-lead ECG analysis revealed that the duration of P waves was significantly longer in the LVH-affected rhesus macaques (44 ± 10.75 ms) compared the controls (33 ± 9.49 ms) (*P* = 0.026). The duration of the QRS complex was also significantly longer in LVH-affected rhesus macaques (69 ± 13.7 ms) than the controls (51 ± 9.94 ms) (*P* = 0.0035). Although the R wave amplitude was not significantly different between the groups, an absolute QRS complex amplitude was significantly larger in the LVH-affected rhesus macaques (1.5 mV; 1.15–2 mV) compared to the controls (0.925 mV; 0.8–1.1 mV) (*P* = 0.0034). Other ECG patterns were not significantly different between those in the LVH and control groups (Table 1).

**Table 1:** The rhythm analysis was completed and the HR, RR interval, and the amplitude/duration of P waves and QRS complexes, as well as the duration of PQ and QT intervals were obtained from the standard 10 s 6-lead ECG parameters.

**ECG parameters**	**LVH**	**Control**	***P*-value**
HR (bpm)	143.5 (±19.93)	134.8 (±19.83)	0.34
PR-interval (ms)	98 (±14.76)	96 (±12.65)	0.75
P amplitude (mV)	0.175 (0.1–0.3)	0.1 (0.1–0.15)	0.1
P duration (ms)	44 (±10.75)	33 (±9.49)	0.026^*^
R amplitude (mV)	1.25 (0.79–1.69)	0.83 (0.74–0.93)	0.12
Absolute QRS amplitude (mV)	1.5 (1.15–2)	0.93 (0.8–1.1)	0.0034^*^
QRS duration (ms)	69 (±13.7)	51 (±9.94)	0.0035^*^
QT (ms)	227 (20.58)	226 (26.33)	0.93
QTc (ms)	302.2 (±19.29)	294.4 (±27.53)	0.47
MEA (degree)	60 (45–75)	60 (60–63.75)	0.95

The ECG parameters were compared between the LVH and control groups. The values were listed as mean ± a standard deviation or median with an interquartile range. HR, heart rate; QTc, corrected QT interval; MEA, mean electrical axis; LVH, left ventricular hypertrophy.

* *P < 0.05*.

### Analysis of 20-h Holter Monitoring

**Figure 5:**
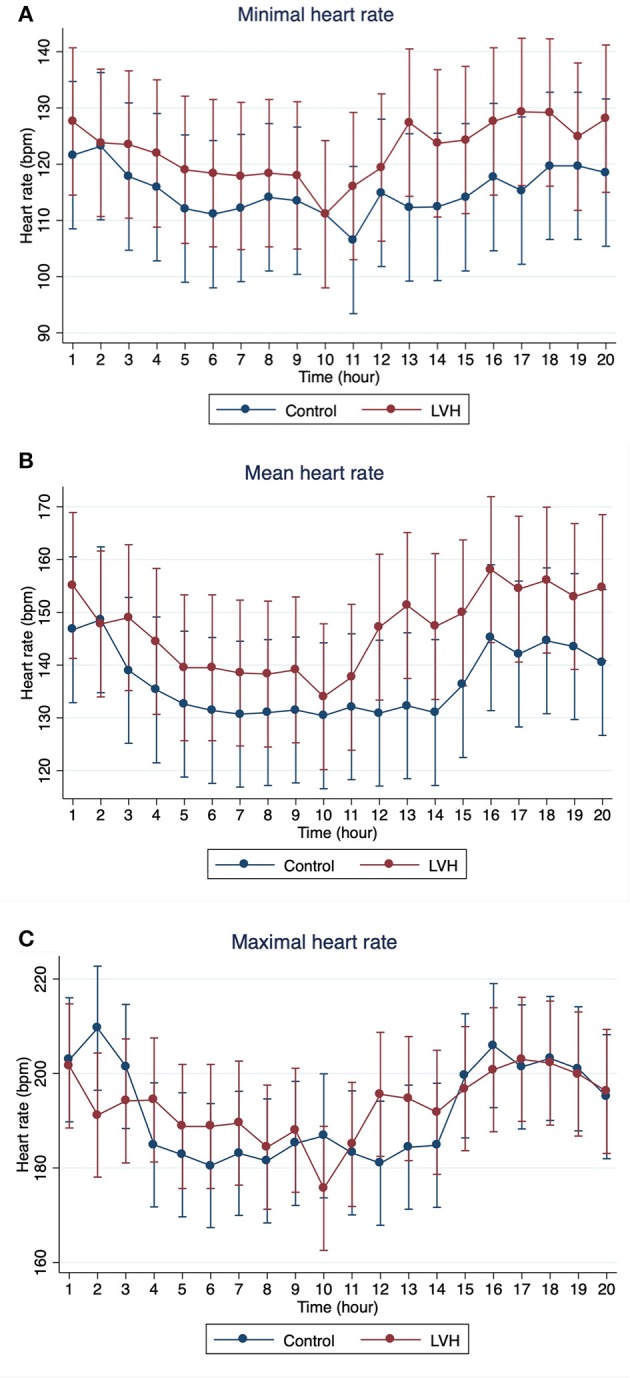
The means and 95% confidence intervals of **(A)** minimal HR, **(B)** mean HR, **(C)** maximal HR for every 1 h over 20-h Holter analysis were noted with a red solid line for the LVH group and a blue solid line for the control group.

**Figure 6:**
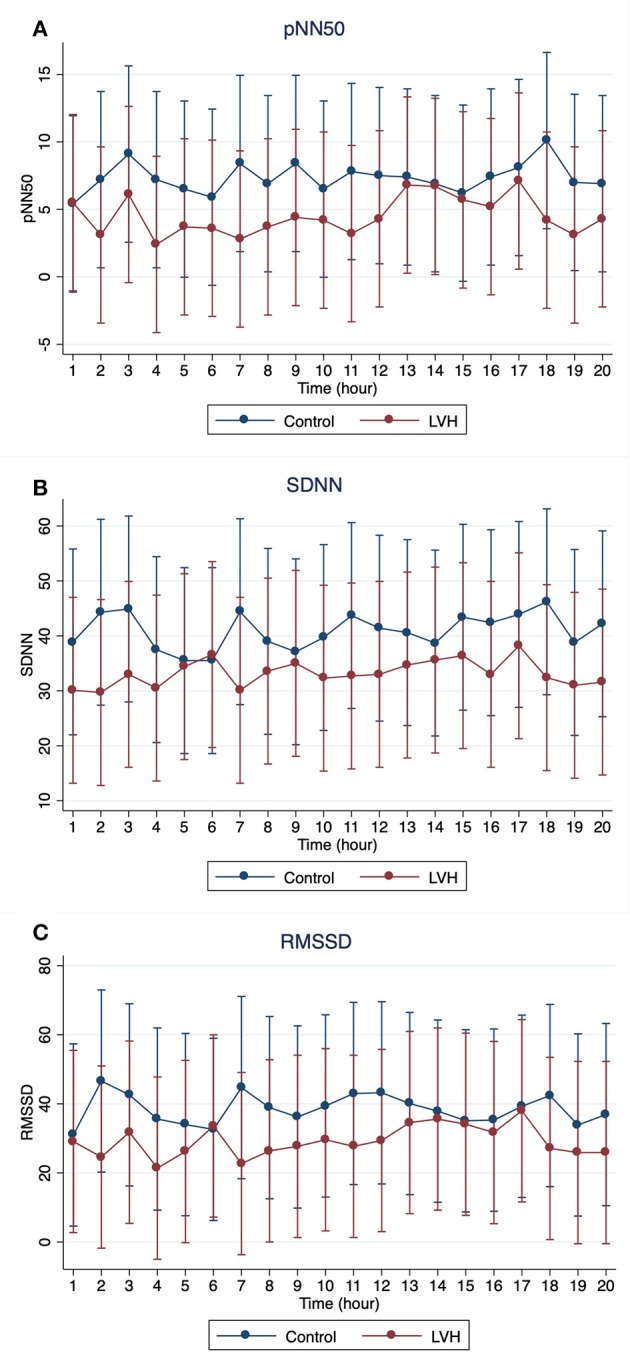
The means and 95% confidence intervals of **(A)** pNN50, **(B)** SDNN, **(C)** RMSSD for every 1 h over 20-h Holter analysis were noted with a red solid line for the LVH group and a blue solid line for the control group.

The Holter monitoring devices were uneventfully placed for all 20 rhesus macaques under sedation with ketamine, and all devices successfully recorded 24-h of ECG data (Figure 4). No significant differences were identified in the frequency and/or complexity of ventricular or supraventricular arrhythmias between LVH and control groups (Table 2). The results of mean time-domain HRV parameters, HR and corrected QT-interval (QTc-interval) over 20 h are shown in Figures 5, 6. No significant differences in these parameters between LVH and control groups were observed (Table 3). Repeated measure assessment via linear mixed model was also performed to determine the effect of LVH status, time and the mixed effects of LVH status and time on HR, HRV parameters, and QTc-intervals (Table 4). There were no statistically significant effects with regards to LVH status on HR, HRV parameters and/or QTc-intervals, whereas significant effects of time to the minimal HR (*P* < 0.0001), mean HR (*P* < 0.0001), maximal HR (*P* < 0.0001), triangular index (P < 0.0001), and the mean QTc-interval (*P* = 0.019) were noted. The significant mixed effect of LVH status and time were noted on maximal HR (*P* = 0.0046). Furthermore, the correlations analysis was performed between the parameters obtained from the Holter analysis, left ventricular wall thickness (IVS and LVPW), age and weight of the animals. There were no significant correlations among these variables, except for between the body weight and maximal HR [*P* = 0.017 (95% CI: −6.15 to −0.72)].

**Table 2:** Frequencies and complexities of ventricular and supraventricular arrhythmias were obtained from the 20-h Holter monitoring and compared between the LVH and control groups.

**Arrhythmias**	**LVH**	**Control**	***P*-value**
Total ventricular arrhythmia (beats per 20 h)	0 (0–3.5)	2 (0–14)	0.33
Class 1	0 (0–3.5)	2 (0–11.25)	0.25
Class 2	0 (0–0)	0 (0–0)	
Class 3	0 (0–0)	0 (0–0)	
Class 4	0 (0–0)	0 (0–0)	
Total supraventricular arrhythmia (beats per 20 h)	0 (0–0)	0 (0–2.5)	0.087
Class 1	0 (0–0)	0 (0–2)	0.087
Class 2	0 (0–0)	0 (0–0)	
Class 3	0 (0–0)	0 (0–0)	
Class 4	0 (0–0)	0 (0–0)	

*The values were listed as a median and an interquartile range. bpm, beat per minute; LVH, left ventricular hypertrophy*.

**Table 3:** HR, HRV parameters, and QTc-intervals were obtained from 20-h Holter study and compared between LVH and control groups.

**HRV parameters**	**LVH**	**Control**
Minimal HR	122.5 (±17.06)	115.2 (±21.68)
Mean HR	146.8 (±16.69)	136.8 (±23)
Maximal HR	193.1 (17.84)	191.9 (17.27)
pNN50	2.05 (1.76–4.91)	2.4 (0.13–8.84)
RMSSD	21.88 (18.61–30.81)	21.75 (14.83–34.33)
SDNN	27.28 (21.5–38.76)	35.03 (21.58–41.38)
SDANN	28.43 (±11.25)	29.76 (±12.86)
Triangular index	8.6 (7.55–12.3)	10.05 (6.288–11.99)
Minimal QTc	358.2 (±23.79)	366.8 (±366.8)
Mean QTc	397.7 (±26.53)	393.3 (±31.01)
Maximal QTc	442.2 (±34.23)	433.2 (±33.66)

*The values were listed as mean ± a standard deviation or median with an interquartile range. pNN50, the proportion obtained by dividing NN50 by the total number of NN intervals; RMSSD, the square root of the mean squared differences of successive NN intervals; SDNN, the standard deviation of all NN intervals; SDANN, the standard deviation of the average NN intervals; QTc, corrected QT interval; LVH, left ventricular hypertrophy*.

**Table 4:** Repeated measure analysis with linear mixed model were performed to determine the effects of the LVH status and time over 20 h to the heart rate, HRV parameters and the QTc-intervals.

**HRV parameters**	**LVH status**	**Time**	LVH status^*^time
	***P*-value**	***P*-value**	***P*-value**
Minimal HR	0.4	< 0.0001^*^	0.36
Mean HR	0.27	< 0.0001^*^	0.32
Maximal HR	0.88	< 0.0001^*^	0.0046^*^
pNN50	0.50	0.67	0.27
RMSSD	0.60	0.77	0.41
SDNN	0.51	0.67	0.27
SDANN	0.81	0.10	0.46
Triangular index	0.68	< 0.0001^*^	0.54
Min QTc	0.44	0.084	0.074
Mean QTc	0.75	0.019^*^	0.71
Max QTc	0.55	0.64	0.72

pNN50, the proportion obtained by dividing NN50 by the total number of NN intervals; RMSSD, the square root of the mean squared differences of successive NN intervals; SDNN, the standard deviation of all NN intervals; SDANN, the standard deviation of the average NN intervals; QTc, corrected QT interval; LVH, left ventricular hypertrophy.

* *P < 0.05*.

## Discussion

One of the most common clinical manifestations of HCM in young adults and LVH in rhesus macaques is SD often without any preceding clinical signs (1, 8). Known risk factors of developing SD in human patients with HCM include severe supraventricular and ventricular arrhythmias (33). However, identification of HCM patients with high risk of SD is still challenging. In rhesus macaques, an early identification of LVH is crucial for the management of the disease in the research colony at CNPRC. Ante-mortem evaluations of these rhesus macaques using echocardiographic and short-term and long-term ECG evaluations could be useful tools to identify rhesus macaques in the early stages of the disease prior to SD events. In addition, evaluations of short-term and long-term ambulatory ECG monitoring including HRV analysis might give us further phenotypic characterizations of LVH in rhesus macaques to establish them as a non-human primate model for the future direct translational approaches of human HCM and SD.

Standard short-term ECG monitoring often shows abnormal ECG patterns and morphologies in human HCM patients (34). These common ECG changes with HCM include large and prolonged QRS complexes as well as deep narrow Q-waves (7, 34, 35) Importantly, these abnormalities can be present even in human HCM patients before developing obvious LVH (6, 7). In the present study, the standard 10 s 6-lead ECG monitoring identified significantly longer duration of the QRS-complex and P-wave in the rhesus macaques with LVH compared to the controls (Table 1). This finding further supports that the alteration in ECG patterns is consistent with the presence of a hypertrophic left ventricular wall with delayed electrical conduction. An increased absolute amplitude of the QRS-complex in spite of a non-significant increase of R-wave amplitude confirms the presence of a prominent Q-wave, which is known to be associated with asymmetric HCM in human patients (35).

The significantly prolonged P-wave is generally observed in human patients with left atrial enlargement due to increased filling pressure in the left ventricle secondary to hypertrophic cardiomyopathy (36). In addition, altered LA function and electrophysiology can also result in prolonged duration of a P-wave (36). In the rhesus macaques with LVH enrolled to the present study, no significant left atrial enlargement was noted on the echocardiographic examination compared to that of the control group. The prolonged P-wave in the LVH rhesus macaques could thus be related to an altered left atrial function and/or electrophysiology. To determine the exact mechanisms of the prolonged P-wave in the rhesus macaques with LVH in the present study, further investigations including histopathological examination of the left atrial wall and advanced imaging such as cardiac magnetic resonance image would be necessary. These findings support the idea that a short-term ECG analysis could be utilized as a part of ante-mortem screening examinations for LVH in the rhesus macaque colony. In human HCM patients, however, these changes in the ECG patterns are known to be unrelated to the severity of HCM and they do not predict SD in these patients (37, 38). Therefore, short-term ECG evaluation may not help prognosticate LVH-affected rhesus macaques or predict SD due to malignant arrhythmias. Further investigations are necessary to identify the prognostic value for these findings.

Twenty-hour Holter monitoring did not reveal significant differences in the frequency and complexity of ventricular and supraventricular arrhythmias between LVH and control groups (Table 2). This finding might be due to the effect of wearing the jacket and being confined individually in the cage during the study period increasing the effects of sympathetic stimulation. Nevertheless, subjectively large reductions in heart rate within the initial couple hours of monitoring indicates the effects of sympathetic drive due to stress is most likely negligible after the first 4 h of recording. Alternatively, this stress-induced condition could represent a better situation for assessing the true frequency and complexity of cardiac arrhythmias since these scenarios may precipitate arrhythmogenesis and represent a physiologic stress test. The findings of the present study indicate that the frequency and complexity of ventricular and supraventricular arrhythmias are not significantly associated with the presence of LVH in rhesus macaques. Further studies are certainly necessary to determine the risk stratifications of SD due to LVH in rhesus macaques.

Heart rate variability decreases as vagal tone is overridden by sympathetic stimulation, often due to cardiac disease and critically ill conditions (27, 39). In humans, HRV has been used as a tool to evaluate sympatho-vagal balance in patients with cardiac diseases, and multiple studies revealed decreased HRV as an independent risk factor for higher mortality and cardiac-related SD in these patients (15, 40, 41). HRV has also been investigated in veterinary patients including dogs (42–47), cats (48, 49), horses (50, 51), and cattle (52, 53), but to the author's knowledge, the present study is the first study of the HRV evaluation in rhesus macaques with cardiac disease. In human HCM patients, it is controversial if the sympathetic or parasympathetic tone predominates in different stages and types of HCM, and decreased HRV in patients with HCM has been observed in several previous studies but is not consistent (18, 19, 54, 55). In addition, decreased HRV failed to predict the risk of SD in adult HCM patients, although a weak association was detected between the risk of SD and decreased HRV in children with HCM (18, 37, 38).

In the present study, no significant effect of the LVH status on the HR, HRV parameters or QTc-intervals were noted, whereas the HR, triangular index, and mean QTc-intervals changed significantly over the course of 20 h (Tables 3, 4). Interestingly, the graphs obtained from the repeated measure analysis with linear mixed model revealed the trend of increased HR and decreased HRV parameters though they were not significantly different (Figures 5, 6, Supplementary Figure 1). This might be suggesting that the results of the present study are affected by Type II error and that a larger sample size is required for future analyses to obtain the statistically significant differences. Nevertheless, this result is consistent with the results of some HRV analysis studies in adult HCM patients, and the present study suggests that HCM might not necessarily reduce the HRV in both adult humans with HCM and rhesus macaques with LVH. In addition, diminished HRV in human HCM patients were reported to be associated with left atrial enlargement, presumably due to the severe form of HCM (21). None of LVH-affected rhesus macaques in this study had left atrial enlargement, and this might be another reason for the lack of HRV differences in rhesus macaques with LVH compared to the control rhesus macaques. Vagal parasympathetic stimulation can also be caused by the presence of concurrent diseases including central nervous, respiratory and gastrointestinal diseases which are not uncommon to concurrently occur with cardiac diseases including HCM. In the present study, the influences of concurrent diseases are less likely since these rhesus macaques did not show any clinical signs of these systemic diseases and were deemed to be healthy before entering the present study based on their frequent colony health checks. Frequency-domain HRV was not assessed in the present study. Indeed, several studies in human patients with and without HCM showed strong correlation between frequency- and time-domain HRV variables obtained over 24-h period, and thus the results of the frequency-domain analysis is likely to be equivalent to those of the time-domain analysis obtained in the present study (18, 21, 27).

This study has a few limitations. First, the small sample size may result in Type II error. The power calculation was performed based on the frequencies of arrhythmias with and without HCM in human patients. It is possible that the healthy and LVH-affected rhesus macaques may not have similar frequencies of arrhythmias as human patients. Furthermore, because of the trend to have lower HRV parameters in rhesus macaques with LVH compared to controls, a follow-up study with larger sample size might reveal significant associations between LVH status and HRV parameters. In addition, the experimental designs including ketamine administration as a sedative and confinement of the rhesus macaques wearing the Holter device and jacket could mask the actual differences of the arrhythmias and HRV parameters between LVH and control groups. Therefore, follow-up studies, longer washout periods for ketamine and additional acclimation time for rhesus macaques to acclimate wearing the jacket before starting the Holter recording should be considered.

In the present study, analysis of the ECG patterns, HR, frequency and complexities of arrhythmia, and HRV parameters over 20 h using a Holter monitoring device are documented to be feasible in rhesus macaques. Short-term ECG monitoring to assess ECG patterns, especially the duration of P-waves and QRS-complexes, as well as the QRS complex amplitude might aid in diagnosis of LVH in rhesus macaques. However, frequency and complexities of arrhythmias and HRV parameters were not significantly different in rhesus macaques with and without LVH. This finding might be due to inconsistent stimulation of sympathetic and parasympathetic nervous systems in humans with HCM and rhesus macaques with LVH. The experimental design of the present study might have masked true associations between arrhythmia and HRV parameters and LVH. Further studies are necessary to characterize ECG changes and autonomic balance in rhesus macaques with more severe LVH using a larger number of patients with longer durations of the experiments.

## Author Contributions

JS, RR, and JR conceived the research. JS, RR, and YU selected the study subjects. YU, TS, and JS collected the data. YU, TS, AW, and EO analyzed the data. YU wrote the manuscript. All the authors contributed to the revision of the manuscript and approved the final version of the manuscript for submission.

### Conflict of Interest Statement

The authors declare that the research was conducted in the absence of any commercial or financial relationships that could be construed as a potential conflict of interest.

## Supplementary Material

Supplementary Figure 1 The means and 95% confidence intervals of **(A)** SDANN, **(B)** Triangular index, **(C)** mean QTc, **(D)** minimal QTc, and **(E)** maximal QTc for every 1 h over 20-h Holter analysis were noted with a red solid line for the LVH group and a blue solid line for the control group.

## Abbreviations

CNPRC: california national primate research center
ECG: electrocardiography
HCM: hypertrophic cardiomyopathy
HR: heart rate
HRV: heart rate variability
IVSd: interventricular septal wall thickness during diastole
LVH: left ventricular hypertrophy
LVPWd: left ventricular posterior wall thickness during diastole
NN: normal-to-normal
RMSSD: square root of the mean squared differences of successive NN intervals
SD: sudden death
SDANN: standard deviation of the average NN intervals
SDNN: standard deviation of all NN intervals
QTc-interval: corrected QT-interval.
