# Supplementary figures and images for: Heart Rate and Heart Rate Variability of Rhesus Macaques (Macaca mulatta) Affected by Left Ventricular Hypertrophy
Source: Front Vet Sci. 2019 Jan 22;6:1. doi: 10.3389/fvets.2019.00001 (PMC6349711; doi:10.3389/fvets.2019.00001)

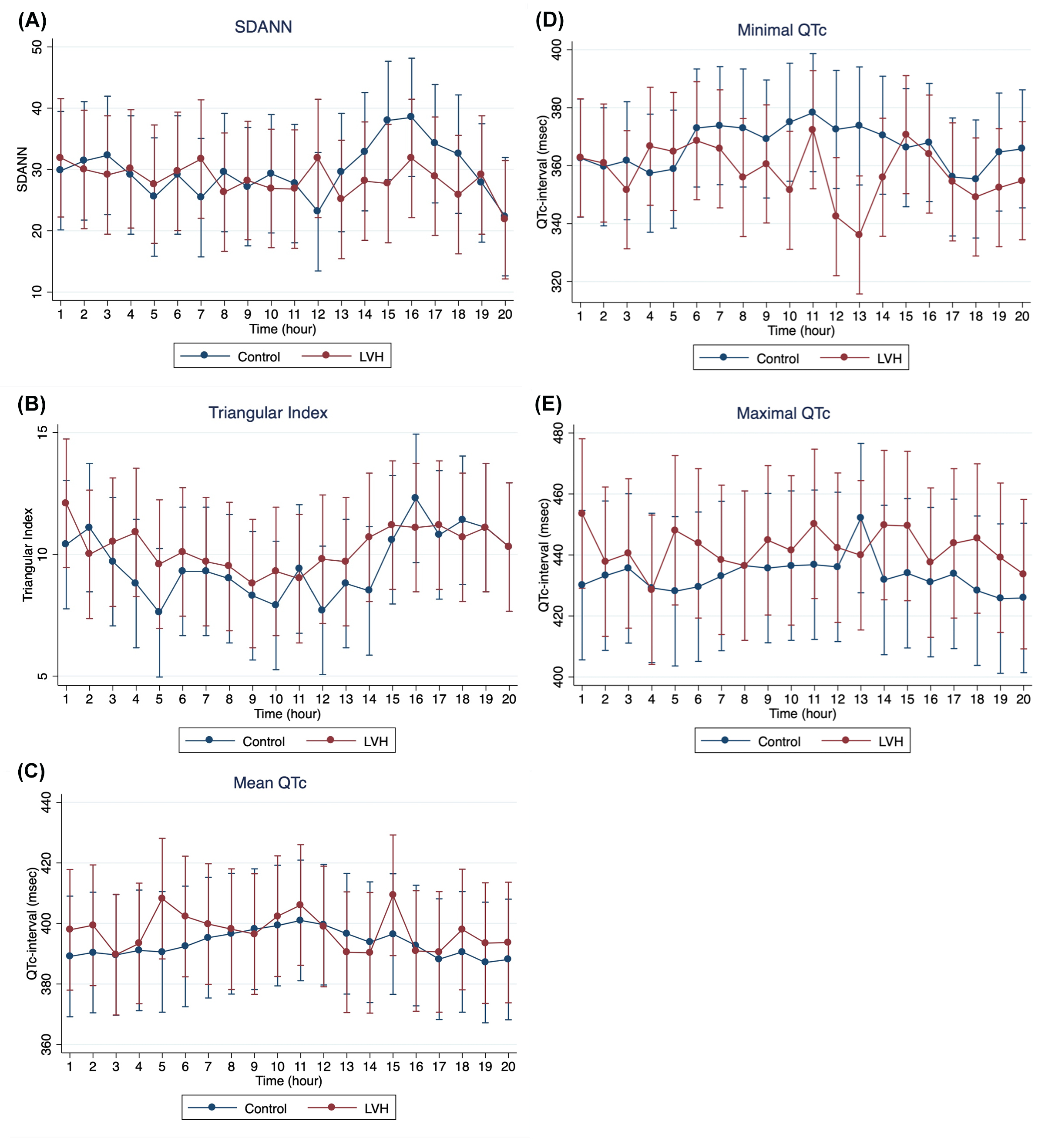

Supplement: Supplementary Figure 1 — The means and 95% confidence intervals of (A) SDANN, (B) Triangular index, (C) mean QTc, (D) minimal QTc, and (E) maximal QTc for every 1 h over 20-h Holter analysis were noted with a red solid line for the LVH group and a blue solid line for the control group. [file Image_1.jpg]
